# Biological Control and Mitigation of Aflatoxin Contamination in Commodities

**DOI:** 10.3390/toxins13020104

**Published:** 2021-02-01

**Authors:** Ferenc Peles, Péter Sipos, Szilvia Kovács, Zoltán Győri, István Pócsi, Tünde Pusztahelyi

**Affiliations:** 1 Institute of Food Science, Faculty of Agricultural and Food Sciences and Environmental Management, University of Debrecen, Böszörményi str. 138, H-4032 Debrecen, Hungary; pelesf@agr.unideb.hu; 2 Institute of Nutrition, Faculty of Agricultural and Food Sciences and Environmental Management, University of Debrecen, Böszörményi str. 138, H-4032 Debrecen, Hungary; siposp@agr.unideb.hu (P.S.); gyori.zoltan@unideb.hu (Z.G.); 3 Central Laboratory of Agricultural and Food Products, Faculty of Agricultural and Food Sciences and Environmental Management, University of Debrecen, Böszörményi str. 138, H-4032 Debrecen, Hungary; kovacs.szilvia@agr.unideb.hu; 4 Department of Molecular Biotechnology and Microbiology, Institute of Biotechnology, Faculty of Science and Technology, University of Debrecen, Egyetem Square 1, H-4032 Debrecen, Hungary; pocsi.istvan@science.unideb.hu

**Keywords:** biocontrol, aflatoxin, pre-harvest, post-harvest, non-aflatoxigenic, ruminal

## Abstract

Aflatoxins (AFs) are toxic secondary metabolites produced mostly by *Aspergillus* species. AF contamination entering the feed and food chain has been a crucial long-term issue for veterinarians, medicals, agroindustry experts, and researchers working in this field. Although different (physical, chemical, and biological) technologies have been developed, tested, and employed to mitigate the detrimental effects of mycotoxins, including AFs, universal methods are still not available to reduce AF levels in feed and food in the last decades. Possible biological control by bacteria, yeasts, and fungi, their excretes, the role of the ruminal degradation, pre-harvest biocontrol by competitive exclusion or biofungicides, and post-harvest technologies and practices based on biological agents currently used to alleviate the toxic effects of AFs are collected in this review. Pre-harvest biocontrol technologies can give us the greatest opportunity to reduce AF production on the spot. Together with post-harvest applications of bacteria or fungal cultures, these technologies can help us strictly reduce AF contamination without synthetic chemicals.

## 1. Introduction

Aflatoxins (AFs) are furanocoumarin derivative mycotoxins biosynthesized by *Aspergillus* species, among which *Aspergillus flavus*, *A. parasiticus*, *A. nomiae*, and *A. pseudotamarii* are regarded as primary producers [1]. AFs can contaminate various products (e.g., cereals, oilseeds, nuts, spices, fruits, dried fruits, and milk) [2]. Regarding the background of the AFs production, the regulation of the AF gene clusters is still studied intensively [3]. The ecology of the AF-producer fungi is remarkably complex, and most likely, interactions of the producer fungal species with host plants and the soil micro- and macrobiota should be considered in detail to reach a deeper understanding of the reasons for AF production [4]. 

AFs are among the most dangerous compounds affecting the physiological processes of animals and humans disadvantageously. AFs are mutagenic, teratogenic, genotoxic, and carcinogenic under long-term exposure [5,6,7]. AFs may enter the feed and food chain at any time point from pre-harvest to human consumption [6,8,9]. These toxins are typically absorbed from the gut in both animals and humans and transferred into different body parts, where they can be chemically bonded or modified. Pathological dysfunction of the liver and kidneys, the gastrointestinal tract, and the immune and reproductive systems have been reported in both humans and livestock under AF exposition [5,6]. AF derivatives like aflatoxin M1 (AFM1) are eventually excreted in animals and cause contamination of milk and milk products [10,11,12,13]. At the same time, AFM1 can be produced in fungal contamination [14]. It may disturb the early development of embryos after getting through the placenta [6,15]. In breastfeeding, AFM1 is threatening even human newborns [12,13]. The direct contamination of fermented milk products like cheese by AF-producer molds and their metabolites has also been described in many cases [16]. 

Researchers work actively to prevent mycotoxin formation and carry-over because the dangerous effects of AFs on livestock and human health cannot be underestimated. Prevention methods and protocols set into operation pre- and postharvest, for example, good agricultural and manufacturing practices (like deep plowing and grain sorting) and appropriate storage conditions (cold, dry environment), are regarded as the best choices to reduce the AF contamination in feed and food. However, these are not always possible [7].

AFs do not decompose quickly because of their remarkable stability [17,18]. Therefore, preventing the growth of AF-producer fungi and detoxification of contaminated feeds and foods is critical. Several chemical (e.g., fungicides), physical (e.g., radiation), and biological detoxification methods were investigated and applied [19]. The chemical and physical methods may lead to nutrient reduction and sensory property changes, and mount food safety problems [20,21]. The biological prevention methods take advantage of the adverse effects of selected microorganisms, including bacteria, yeasts, and nontoxigenic molds, on the growth and AF production of toxigenic fungal strains. The adverse effects of these techniques are based on space and nutrient competitions (competitive exclusion), or biological interactions like antibiosis. The microorganism-based biological control technologies can be reasonable solutions for controlling and reducing pre- and post-harvest AF contamination in crops and food products. Notably, such biological control technologies have been commercialized and are available on the market [22,23,24].

In this review, special attention is paid to reducing AFs by biological methods in feed and food pre- and post-harvest and emphasizing promising novel and innovative approaches and technologies.

## 2. Document Analysis

The aim was to gain information on AF and *Aspergillus* mitigations work with any microbial or biological agents. Therefore, different online libraries (PubMed, Google Scholar, Science Direct, and Mendeley) were searched for the following terms and phrases: “aflatoxin degradation”, “aflatoxin binding”, “aflatoxin AND ruminal degradation”, “non-aflatoxigenic *Aspergillus*”, “atoxigenic *Aspergillus*”, “aflatoxin AND biocontrol”, “*Aspergillus* AND biofungicide”, and “competitive exclusion AND *flavus*”. The findings were ordered as ruminal, pre-, and post-harvest processes to see all natural and biotechnological possibilities and attempts. Investigations using viable cells in milk or phosphate-buffered saline as a medium were only considered for a clearer comparison of the effects on AF binding or degradation. 

## 3. Ruminal Detoxification of Aflatoxins

It was common to feed ruminants with the fodder of AFs contamination because it was known that ruminal microorganisms could detoxify mycotoxins. However, the scientific literature is still diverse about AFs’ fate in ruminal animals. In 1978, Engel and Hagemeister [25] reported 42% degradation of aflatoxin B1 (AFB1) in vitro applying rumen fluid. The effectiveness of the degradation depended on the animal species and the fodder [26]. The resistance of AFs to ruminal degradation may be caused by the strong inhibitory effect of AFs on rumen microorganisms. It was shown that at high concentration ranges (5 and 10 µg mL^−1^), AFB1 completely inhibited many ruminal bacteria [27] and harmed ruminal fermentation parameters in vitro [28,29]. Moreover, the fodder composition also impacts the degradation success since the high-starch diet increased the available AFB1 and ochratoxin [30]. Some authors disputed that rumen fluid affected AFB1 in contrast to deoxynivalenol, T-2, or zearalenone mycotoxins [31,32].

## 4. Pre-Harvest Biocontrol

### 4.1. Pre-Harvest Biocontrol by Competitive Exclusion

A biocontrol technology relying on endemic non-aflatoxigenic *Aspergillus flavus* strains as competitors of AF-producer fungi is becoming widespread, as it is remarkably successful, cost-effective, and environmentally friendly.

Carefully selected endemic non-aflatoxigenic genotypes can efficiently reduce AF contamination when applied before plant flowering. Hruska et al. [33] demonstrated via competitive exclusion that the decreased AF level was positively correlated to the low population of the toxigenic *A. flavus* under treatment, where the biocontrol *A. flavus* strain showed increased propagation and colonization. Recent studies demonstrated that biocontrol non-aflatoxigenic strains reduced AF concentrations in treated crops by more than 80% under both field and storage conditions [34,35,36]. In Argentina, a native atoxigenic *A. flavus* strain showed a remarkable biocontrol potential on peanuts, and the pre-harvest application of the biocontrol agent had a carry-over effect and protected peanuts under storage conditions [37]. The main criterion in the strain selection is the high colonization ability of the atoxigenic strain [38]. Researchers showed there was no competitive advantage of the selected atoxigenic fungal strains over the toxigenic strains and could exclude the AF role in peanut infections of *A. flavus* and *A. parasiticus* [39]. Moreover, these strains were equally applicable to peanut and maize host plants [40]. Multi-strain biocontrol products of non-aflatoxigenic *A. flavus* showed both immediate and long-term beneficial effects under different conditions compared with single-strain products [34,38,41,42]. However, the employed biocontrol product’s efficacy depends on several factors, including inoculum rate, formulation, application of herbicide, the soil’s temperature, and the availability of water and substrate [7,43]. Extrolites (e.g., volatile organic carbons and secondary metabolites) secreted by the biocontrol strains may also increase the efficacy of the control, and future biocontrol strategies may take advantage of these not-yet-characterized compounds [44]. *Aspergilli* have an outstanding secondary metabolite production potential, and it includes aflatrems, aflavarins, aflavinines (only in sclerotium producers), cyclopiazonic acids, kojic acid, and other potentially toxic compounds besides AFs [1]. The possible overproduction of any health hazard metabolites, like cyclopiazonic acid, should be carefully checked in the chosen strains. The potential non-aflatoxigenic biocontrol strains should be real atoxigenic ones [45] without any production of, at least, the known toxic molecules under field conditions. 

Formulation and application strategies of the biocontrol agent are of paramount importance to reach the required efficacies. Solid-state fermented rice, encapsulated *A. flavus* fungal conidia in an extrusion product (Pesta), pregelatinized corn flour granules [46], rice, cracked barley, intact canola seed [39], and sterile sorghum grain [34] were treated with a spore suspension of nontoxigenic *A. flavus*, *A. parasiticus*, or both. Application of all formulations significantly decreased the AF contamination of peanuts, and the strains were found to be long-term viable on these matrixes. A more significant or consistent AF reduction was recorded with fluid and granular delivery of biocontrol strains of *A. flavus,* and both methods were efficient and cost-effective [47]. However, granular delivery has not gained ground in field applications because of the difficulties with applying a granular product through the canopy in the reproductive stage of the maize development. Water-dispersible granule formulations of biocontrol strains can also be useful. Weaver et al. [48] demonstrated that their new formulation with higher wettability and rapid dispersion resulted in more than 49% decrease in AF contaminants in all treatments with the Alfa-Guard biocontrol strain (*A. flavus* NRRL 21882). In another study, Accinelli et al. [49] evaluated an application method for the biocontrol strains on leaf. The preparation proved to be adherent, the biocontrol strain showed good leaf surface colonization, and it reduced AF level of the kernels by up to 80%–90%. In comparison, the number of AF-producing *A. flavus* in the soil was not changed significantly [49]. 

An exciting novel approach was also published by Accinelli et al. [50], who used a starch-based bioplastic formulation to coat corn kernels, which contained two conventional pesticides and spores of the non-aflatoxigenic *A. flavus* NRRL 30797 strain. Significantly, the additives did not affect the kernel germination adversely or the seedlings’ growth, while the AF contamination was reduced.

The competitive exclusion method was first set in the USA, and similar technologies working with endemic strains have been developed in several African countries [34,41,42,51,52] (Table 1). A non-aflatoxigenic strain (*A. flavus* NRRL 21882) under the commercial name Afla-Guard (Syngenta, Basel, Switzerland), which is marketed in the USA, has been used successfully on maize, groundnuts, pistachios, and cottonseed for many years [36,40]. A mixture of four endemic non-aflatoxigenic *A. flavus* strains called Aflasafe (Ibadan, Nigeria) also has been used on maize and groundnuts in various African countries, with an AF contamination reduction rate of 80–99% [35,41,51,53,54,55,56]. A commercial product AF-X1 (*A. flavus* MUCL54911, Pioneer Hi-Bred, Italy) is applied in Italy to prevent AF contamination [57].

### 4.2. Pre-Harvest Biocontrol by Microbial Biofungicides

Besides atoxigenic *A. flavus* biocontrol strains, some other promising biocontrol agents are emerging against AF-producer molds. For example, a *Trichoderma harzianum* strain was applied to restrict *A. flavus* contamination, with 57% and 65% reduction on AF levels in groundnut [58] and in sweet corn [59], but there were no commercialized products found against *A. flavus* [60]. However, Lagogianni and Tsitsigiannis [24] evaluated six biofungicides/stimulants (Botector^®^ (*Aureobasidium pullulans* as anti-*Botrytis* agent; bio-ferm GmbH, Getzersdorf, Austria), Mycostop^®^ (*Streptomyces griseoviridis* as anti-*Fusarium*, *Phytophthora*, *Alternaria,* and *Pythium* agent; Verdera Oy, Espoo, Finland); Serenade Max^®^ (biofungicide, bactericide with *Bacillus subtilis* QST 713; Bayer, Auckland, New Zealand), Trianum^®^ (*Trichoderma harzianum* TT-22 as biofungicide against *Pythium*, *Rhizoctonia*, *Fusarium*, and *Sclerotinia*; Koppert Biological Systems, Berkel en Rodenrijs, The Netherlands); Vacciplant^®^ (biofungicide containing laminarine; Helena Agri-Enterprises, LLC, Collierville, TN, USA) and zeolite inorganic adsorbent) and found most of them useful in reducing *A. flavus* conidiospores and AFB1 production in vitro. Mycostop^®^ and Botector^®^ treatments decreased (43%) the AFB1 content of maize kernels. 

## 5. Post-Harvest Management of Aflatoxin Contamination

The effects of bacteria and yeasts have also been studied extensively to reduce already manifested AF contamination [4,5,7]. Biological detoxification by microorganisms relies on the binding and transformation of AFs into less toxic metabolites by microbial biomass [4,5,21]. These post-harvest methods are needed as, despite the encouraging results, pre-harvest biocontrol methods have their drawbacks. Interactions in the microbiota are in a flux state, even at the strain level, and the biocontrol effect differs under various environmental conditions [61].

### 5.1. Bacteria

Lactic acid bacteria (LABs), for example, *Lactobacillus acidophilus*, *Lactococcus lactis* subsp. *lactis*, *Lactobacillus selangorensis*, *Pediococcus acidilactici*, *Streptococcus thermophilus*, *Weissella confusa*, *Enterococcus avium*, and *Bifidobacterium animalis* subsp. *lactis*, inhibit AF production or remove AFs from feed and food products (Table A1). The sufficient binding of AFs by LAB strains is dependent on the inherent features of the strain, temperature, pH, the matrix itself, and incubation time [20,62]. Asurmendi et al. [63] successfully demonstrated that all LAB strains tested inhibited the growths of aflatoxigenic A. flavus strains and their AFB1 production in brewer’s grains, which is used for feeding pigs. More recently, Saladino et al. [64] tested the beneficial effects of LAB strains on the AF content of bread and found considerable 84%–100% decreases in 4 days. Assaf et al. [65] suggested a method for reducing AFM1 in milk by biofilm-forming probiotic LAB strains. They recorded a 61% reduction of AFM1 by a *Lacticaseibacillus rhamnosus* (formerly *Lactobacillus rhamnosus*) GG biofilm. Such capable bacterial biofilms could be formed on glass, metal, plastic surfaces in a test tube, 96-well plate, or flow cell formats [66]. Wacoo et al. [67] found that the allochthonous LAB species (*L. brevis*, *L. casei*, *L. fermentum*, and *L. plantarum*) isolated from the gut microbiota bound AFs effectively.

The antifungal compounds biosynthesized by LAB can support the reduction of mycotoxin production [20,68]. These compounds usually are organic acids (e.g., acetic, lactic, and propionic acids), carboxylic acids, phenolic compounds, including phenolic acids (benzoic acids, hydroxyphenyl lactic acid, phenyl lactic acid, gallic acid, and tannins), fatty acids (3-hydroxydecanoic acid, coriolic acid, caproic acid, decanoic acid, and ricinoleic acid), volatile organic compounds (e.g., acetoin and diacetyl), cyclopeptides (e.g., cyclo(L-Leu-L-Pro), cyclo(Phe-Pro), cyclo(L-Met-L-Pro), and cyclo(L-Tyr-L-Pro)), ethanol, hydrogen peroxide, proteinaceous compounds, and reuterin [69,70,71,72]. Thus far, the process of the antifungal action of proteinaceous compounds and hydroxy fatty acids has not been elucidated [69]. However, some of them can increase cytoplasmic permeability, which can finally lead to fungal cell death [69]. H_2_O_2_ is well known for its oxidizing potential directly on the lipid components and the cellular membranes’ integrant proteins [69].

Several non-lactic acid bacteria, such as *Bacillus* spp., *Brachybacterium* spp., *Brevundimonas* spp., *Cellulosimicrobium* spp., *Enterobacter* spp., *Escherichia* spp., *Klebsiella* spp., *Mycolicibacterium* spp., *Myxococcus* spp., *Nocardia* spp., *Pseudomonas* spp., *Rhodococcus* spp., *Streptomyces* spp., and *Stenotrophomonas* spp., can also inhibit the growth and AF production of molds (Table A2). For example, probiotic *Enterococcus faecium* M74 and EF031 strains reduced the AFB1 content of aqueous solution by 19–38% [73]. A *Bacillus subtilis* strain also reduced the AFB1 content of contaminated feed and food by 60–95% [74,75]. Moreover, metabolites from *Bacillus subtilis* (bacillomycin D, fengycins A and B, iturin A, mycosubtilin, and plipastatins A and B), *Achromobacter xylosoxidans* (cyclo (L-leucyl-L-propyl)), and *Streptomyces* spp. (blasticidin A, aflastatin A, dioctatin A) are effective inhibitors of AF biosynthesis in vitro and in vivo in crop model systems and field [76]. While the plant-growth-promoting (PGPR) *Pseudomonas aeruginosa* inhibited *A. flavus* growth with only 15% in soil [77]. *Cellulosimicrobium funkei* strains showed outstandingly high (97%) AFB1 biodegradation ability, suggesting that it could be used as a feed additive [78]. *Bacillus* and *Pseudomonas* strains isolated from peanut, pistachio, and maize fields also could be promising new biocontrol agents to reduce the growth of aflatoxigenic fungi and the AF contamination of arable crops [79]. According to Wang et al. [80], the culture supernatant of *Escherichia coli* CG1061 (isolated from healthy chicken cecum) degraded AFB1 by 61.8%, and the strain could colonize the animal gut; therefore, it may also be a suitable candidate for AFB1 detoxification.

Application of active immobilized enzymes of bacterial origin can also be a useful tool to degrade AFs in feeds and foods. In *Mycolicibacterium smegmatis* (*Mycobacterium smegmatis*), two families of F_420_H_2_-dependent reductases were identified that catalyze AF degradation [81,82]. Meanwhile, an AF degradation enzyme (MADE) was also identified from *Myxococcus fulvus* [83].

Bacterial volatile organic compounds are also able to hinder or kill fungal cells. *Alcaligenes faecalis* N1-4 produced several antifungal volatiles and inhibited the growth of *A. flavus* through in vitro testing. GC-MS/MS analysis detected dimethyl disulfide and methyl isovalerate as the two primary compounds in the strain’s volatile organic carbon spectrum [84]. Dimethyl disulfide hindered the germination of conidia and the growth of *A. flavus*. These volatile organic compounds repressed the gene expression of 12 genes in the AF biosynthesis pathway, and eight genes were significantly downregulated [84]. In groundnut, rice, maize, and soybean with high water activity, *A. flavus* infection and AFs contamination were entirely inhibited by *Enterobacter asburiae* Vt-7 volatile organic compounds (phenyl ethyl alcohol and 1-pentanol) [85]. In Vitro, volatile organic carbons from *Streptomyces yanglinensis* 3-10 inhibited growth, conidial germination, asexual sporulation, and expression of AFB1 biosynthesis cluster genes in *A. flavus* and *A. parasiticus*, and, in vivo, reduced the disease symptoms on peanut kernels [86]. The volatile organic carbons suppressed the mycelial growth of more than 15 plant pathogenic fungi and an oomycete organism. Chemicals, including 2-phenyl ethanol, methyl 2-methyl butyrate, and β-caryophyllene, showed activity against *A. flavus* and *A. parasiticus*. Therefore, *S. yanglinensis* 3-10 may become a promising biofumigant in the control *A. flavus* and *A. parasiticus* [86].

Microbial volatile organic carbons are also investigated as plant growth inducers, whose characteristics belong to various groups of chemicals, including alcohols, sulfur compounds, terpenes, ketones, and furans. Microbial volatiles can stimulate growth by modulating hormonal balance, essential nutrients, metabolism, and sugar concentrations. The alterations are coupled mostly to cellular structure and stress response genes [87,88,89].

### 5.2. Yeasts

Several publications have demonstrated that yeasts, for example, *Candida*, *Debaryomyces*, *Pichia*, *Saccharomyces*, *Saccharomycopsis*, *Saccharomycodes*, *Schizosaccharomyces*, *Trichosporon*, and *Zygosaccharomyces* species, inhibited AF production significantly in aflatoxigenic molds (Table A3). It is considered that yeast supplementation (e.g., *Kluyveromyces marxianus* and *Pichia kudriavzevii*) improved AFB1 detoxification in the rumen, reduced the AFM1 content of milk, and improved the performances of dairy cattle [90]. Viable yeast supplement in feed exerts a positive effect on the ruminal environment, total and cellulolytic bacteria, and protozoa [91,92]. Mycotoxin binding of the feed additives, such as bentonite, modified yeast cell-wall extract, or esterified glucomannan, has been shown to reduce the toxic effects of AFB1 in different livestock species by nonspecific binding of the AFs so that they cannot be absorbed in the gastrointestinal tract [93,94,95,96,97]. However, research on the interactions between detoxifying additives and mycotoxins is rare [94,97].

Yeast volatile organic carbons also take part in the hindrance of *A. flavus* growth and AF production [98]. Additionally, yeasts can bind AFs reversibly and rapidly [4,7]. Consequently, the GRAS baker’s yeast *Saccharomyces cerevisiae* can be used safely as a feed additive to mitigate aflatoxicosis in livestock, including both broilers and ruminants [21,99,100,101,102,103,104]. Moreover, *S. cerevisiae* can also be used directly for AF decontamination in food. For example, Shetty et al. [105] reported on the high AF binding capability of *S. cerevisiae* in indigenous fermented foods from Ghana. Furthermore, *S. cerevisiae* and *S. pastorianus* converted the AFB1 content of the raw materials used for wine and beer into a less toxic substance during alcoholic fermentation [106]. Foroughi et al. [107] proposed a unique AF detoxification method for AFM1-contaminated milk. The process relied on baker’s yeast, which had been immobilized on perlite beads, and was suitable to reduce the AFM1 content of all tested milk samples without affecting the milk composition [107]. Such microbial cell immobilization-based methods can be of outstanding practical value and importance when AF decontamination of milk and other drinks is considered. 

### 5.3. Fungal Biomass, Enzymes, and Antifungal Proteins

Glucomannan from fungal cell wall or peptidoglycan and other cell wall polysaccharides can be effective adsorbents for mycotoxins because of their structural complexity. Saki et al. [108] tested the effect of Mycosorb (patented glucomannan-containing yeast product derived from yeast cell wall; Alltech) on broiler performance, organ weight, protein digestibility, plasma characteristics, and metabolizable energy of the diets. They found that Mycosorb was effective in mitigating the harmful effects of AFs in broiler chickens. Haidukowski et al. [109] also demonstrated that nonviable *Pleurotus eryngii* mycelia could be used as a practical and economically feasible feed additive for AFB1 detoxification.

Mycotoxin enzymatic degradation is a simple method for usage in food decontamination. However, for AFs degradation, only some fungal enzyme families are known. For example, the spent mushroom substrate crude extract (SMSE) is a rich source of AF-degrading enzymes (e.g., laccase and Mn-peroxidase), and thereby it is a good candidate for the detoxification of AF-contaminated commodities in the future [110]. An extracellular enzyme from the edible mushroom *Pleurotus ostreatus* showed remarkable AF-degradation activity via cutting the lactone ring of AFB1 [111]. Manganese peroxidase from *Phanerochaete sordida* YK-624 catalyzed the detoxification by the oxidation of AFB1 to AFB1-8,9-epoxide, and the subsequent hydrolysis to AFB1-8,9-dihydrodiol [112]. Another well-studied AF oxidase, the former AF-detoxifizyme from *Armillaria tabescens* (*Armillariella tabescens*) E-20, also attacks the 8,9-unsaturated carbon-carbon bond in AFB1 [113]. Besides enzymes, small-molecular-mass antifungal proteins from filamentous fungi are also characterized by their initiated apoptotic cell death in sensitive fungal pathogens [114,115,116] and regarded as promising future biocontrol agents against many plant-pathogenic and food-deteriorating fungi, including *A. flavus*.

## 6. Conclusions and Future Trends

**Figure 1:**
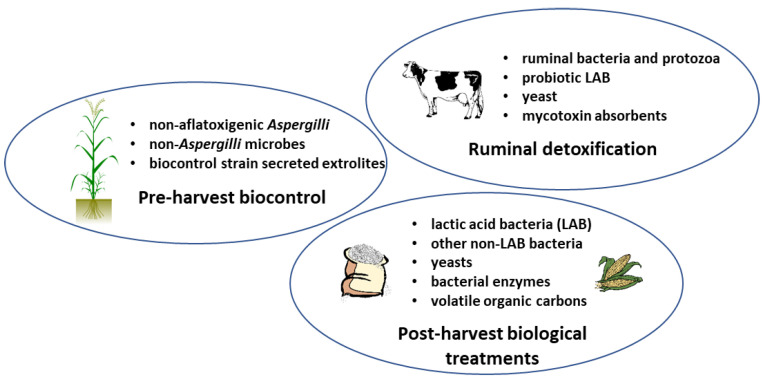
Three typical areas of mitigation of aflatoxins (AF) contamination. Pre-harvest biocontrol methods with non-aflatoxigenic strains of *A. flavus*, other non-*Aspergilli*, and their extrolites are available. In animals fed with contaminated fodder, enteral or ruminal bacteria can degrade or transform AFs to lesser toxic molecules. Besides their beneficial effects on animal health, supplemented probiotic organisms (yeasts and bacteria) can also bind or degrade AFs. In stored food and feed, depending on the water content of the commodity, bacteria, yeasts, or their volatile carbons and enzymes can be used for the AF decontamination in biofilm, immobilized, or encapsulated form.

Natural methods reducing the use of synthetic chemicals represent a promising future trend in AF eliminations. Combinations of physical and biological (natural) methods are expected to improve AF decontamination efficiency, both pre- and post-harvest (Figure 1). 

The most essential requirement for the emerging novel decontamination technologies is that these should not change the physical–chemical properties of the treated feed or food products significantly and no toxic residues of the mycotoxins should be left behind in the decontaminated products. The non-aflatoxigenic, even atoxigenic biocontrol strains are tested mostly for maize, peanuts, groundnuts, pistachios, or cottonseed, while their application in other agricultural sectors like vineyards is also a possibility. When AF contamination occurs in commodities with high water content, like milk, wine, or beer, the application of other technologies like microbial cell immobilization-based methods and enzymatic degradation can have an outstanding practical value and importance. Under the storage of the commodities or in packaging methods, the promising alternatives to synthetic chemicals are the microbial (fungal or bacterial) volatile organic carbons.

Since mammals lack strong natural ruminal or cellular AF degradation, the usual and promising agricultural technology is to help animals with potent probiotic yeasts or bacteria or only their polysaccharides to mitigate the toxic effects. Moreover, pro- or prebiotics are also applied as food supplements. The probiotic supplements have more benefits than the inorganic mycotoxin binders in toxin mitigation, as the microbes have positive physiological effects besides AF binding. Nevertheless, the AF mitigation efficiency is greatly influenced by the nature of the products and the AF contamination level. However, some authors debated the safety of elongated application of LAB or other microbes in food if these cells cannot degrade AFs [117]. Therefore, there is a need to employ starter and probiotic cultures with AF degradation abilities.

It can be stated that there is no general all-purpose decontamination method that could be broadly employed and, hence, one of the main future challenges in this field is to develop new procedures that would support comparable detoxification in a broad spectrum of feed and food matrices. Future research should focus on elaborating these novel technologies and their extensive testing in as versatile feed and food matrices as possible.

## Figures and Tables

**Table 1:** Aflatoxin elimination by non-aflatoxigenic *Aspergillus* strains.

Date	Country	Crop	Non-Aflatoxigenic Strains	Target	Success Rate (%)	References
1994–1997	USA	Maize, peanuts, and cotton	*A. flavus* strains (NRRL 21882 and NRRL 21368), *A. parasiticus* (NRRL 21369)	AFB1, AFB2, AFG1, AFG2 *	66–96	[40]
1996–1997	USA	Peanuts	atoxigenic *A. flavus* (NRRL21368) and *A. parasiticus* (NRRL 21369),	AFB1, AFB2, AFG1, AFG2	86–92	[46]
2004–2006	Kenya	Maize	12 atoxigenic *A. flavus* isolates	AFB1	64–90	[41]
2007–2008	Nigeria	Maize (ACCR-9931-SR)	mixture of four atoxigenic strains of *A. flavus*	AFB1 and AFB2 by *A. flavus* L- and S_BG_-morphotypes, *A. parasiticus* and *A. tamarii*	67–95	[34]
2007–2009	USA	Maize (Pioneer 32R25 hybrid)	Afla-Guard, *A. flavus* NRRL 21882. AF36, *A. flavus* NRRL 18543. *A. flavus* K42	AFB1, AFB2, AFG1, AFG2 by toxigenic *A. flavus* F3W4 (NRRL 30796), K54 (NRRL 58987), NRRL 58976, NRRL 58988, and NRRL 58974	83–98	[40]
2010–2014	Senegal	Groundnut and maize	Aflasafe SN01, mixture of 4 atoxigenic isolates of *A. flavus*	AFB1, AFB2, AFG1, AFG2 by *A. aflatoxiformans*, *A. flavus* L-morphotype, *A. parasiticus*, *A. tamarii*	58–100	[51]
2012–2013	USA	Maize	Afla-Guard, *A. flavus* NRRL 21882. AF36, *A. flavus* NRRL 18543	AFB1 by *A. flavus*, *A. parasiticus*, *A. caelatus*, *A. nomius*, and *A. tamarii*	0–97	[36]
2012–2013	Italy	Maize	AF-X1™, *A. flavus* A2085 and A2321	AFB1	84–95	[57]
2014	Ghana	Maize and groundnut	13 atoxigenic *A. flavus* isolates	AFB1	87–98	[38]

* AFB1, aflatoxin B1; AFB2, aflatoxin B2; AFG1, aflatoxin G1; AFG2, aflatoxin G2.

## Data Availability

Not applicable.

## Appendix A

**Table A1:** Aflatoxin elimination by lactic acid bacteria. We intended to collect data on the viable cells’ bounding properties, usually in PBS or milk at 4 °C.

Aflatoxin	Bacteria	Strain	Toxin Elimination (%)	References
B1	*Bifidobacterium animalis* subsp. *animalis* (formerly *Bifidobacterium animalis*)	CSCC 1941	45.7	[118]
B1	*Bifidobacterium animalis* subsp. *lactis* (formerly *Bifidobacterium lactis*)	E-94508	18	
		CSCC 5094	34.7	
		CSCC 1906	48.7	
B1	*Bifidobacterium longum*	CSCC 5304	37.5	
B1	*Enterococcus faecium*	M74	19.3–30.5	[73]
		EF031	23.4–37.5	
B1	*Lactobacillus acidophilus*	ATCC 4356	48.3	[119]
		E-94507	18.2	[118]
		CSCC 5361	20.7	
		Chr. Hansen, Denmark	56.6	[120]
		CH-2	36	[121]
B1	*Lactobacillus amylovorus*	CSCC 5160	59.7	[118]
		CSCC 5197	57.8	
B1	*Lactobacillus bulgaricus*	Chr. Hansen, Denmark	16.3	[120]
B1	*Lacticaseibacillus casei* (formerly *Lactobacillus casei*) Shirota	YIT 901, commercial	21.8	[119]
		Yakult	98	[122]
B1	*Lacticaseibacillus casei* (formerly *Lactobacillus casei*)	Chr. Hansen, Denmark	22.4	[120]
		Iranian dairy products	43	[123]
B1	*Lactobacillus delbrueckii*	MK9	17.3	[118]
B1	*Lactobacillus delbrueckii* subsp. *bulgaricus*	Australian Starter Culture Research Centre Collection, Werribee, Australia	15.6	[119]
B1	*Limosilactobacillus fermentum* (formerly *Lactobacillus fermentum*)	CSCC 5362	22.6	[118]
		N.A.	≥60	[93]
		Iranian sourdough	61	[123]
		PTCC 1744	99.9	[124]
B1	*Lactobacillus helveticus*	Australian Starter Culture Research Centre Collection, Werribee, Australia	17.5	[119]
		Aki4	30.1	[118]
		Chr. Hansen, Denmark	17.8	[120]
B1	*Lactobacillus johnsonii*	CSCC 5142	30.1	[118]
B1	*Lactiplantibacillus plantarum* (formerly *Lactobacillus plantarum*)	ATCC 8014	29.9	[119]
		E-79098	28.4	[118]
		N.A.	≥40	[93]
		Iranian dairy products	56	[123]
		EMCC-1039	39.2	[121]
B1	*Lacticaseibacillus rhamnosus* (formerly *Lactobacillus rhamnosus*)	DSM 7061	76.5	[119]
		ATCC 53103	54	[125]
			78.9	[119]
		E-97800	22.7	[118]
		Lc 1/3	54.6	
		NRRL B-445	17.2	[121]
B1	*Lactococcus lactis* subsp. *lactis*	Australian Starter Culture Research Centre Collection, Werribee, Australia	59	[119]
		E-90414	31.6	[118]
		Dairy products	54.3	[126]
B1	*Lactococcus lactis* subsp. *cremoris*	Australian Starter Culture Research Centre Collection, Werribee, Australia	26.9	[119]
		MK4	5.6	[118]
		ARH74 (Valio Ltd, Finland)	41.1	
B1	*Lactobacillus selangorensis* (formerly *Paralactobacillus selangorensis*)	N.A.	<39	[93]
B1	*Pediococcus acidilactici*	N.A.	45–59	[93]
B1	*Propionibacterium freudenreichii* subsp. *shermanii* JS	Valio Ltd. Finland	22–82	[125]
				[119]
B1	*Streptococcus thermophilus*	Australian Starter Culture Research Centre Collection, Werribee, Australia	32.7	[119]
		Dairy products	81	[126]
		CH-1	26.9	[121]
B1	*Weissella confuse*	N.A.	15–39	[93]
M1	*Bifidobacterium bifidum*	Bb13	23.48	[127]
		NCC 381	18.11	
		PTCC 1644	99.9	[124]
M1	*Bifidobacterium lactis*	FLORA-FIT BI07 (Danisco Ltd.)	16.89	[128]
M1	*Enterococcus avium*	CTC 469 (Tecnolat, Brazil)	7.36	[128]
M1	*Lactobacillus acidophilus*	NCC 12	16.05	[127]
		NCC 36	22.23	[127]
		NCC 68	14.22	[127]
		LA-5 (Chr. Hansen, Denmark)	95	[62]
M1	*Lactobacillus delbrueckii* subsp. *bulgaricus*	LB340 (Danisco Ltd.)	30.22	[128]
		CH-2 (Chr. Hansen, Denmark)	18.7	[129]
M1	*Lactobacillus gasseri*	ATCC 33323	21.37	[128]
M1	*Lactiplantibacillus plantarum* (formerly *Lactobacillus plantarum*)	CTC368	5.6	[128]
M1	*Lacticaseibacillus rhamnosus* (formerly *Lactobacillus rhamnosus*)	Ezal, France	20.21	[127]
		HOWARU (Danisco Ltd.)	17.13	[128]
M1	*Pediococcus pentosaceus*	TR570 (Tecnolat, Brazil)	8.68	[128]
M1	*Streptococcus thermophilus*	ST-36 (Chr. Hansen, Denmark)	29.42	[129]

**Table A2:** Aflatoxin elimination by non-lactic acid bacteria. We intended to collect data on the viable cells’ bounding properties, usually in PBS or milk at 4 °C.

Toxin	Bacteria	Strain	Toxin Elimination (%)	References
B1	*Bacillus licheniformis*	Thai fermented soybean	74	[130]
B1	*Bacillus stearothermophilus*	N.A.	87	[131]
B1	*Bacillus subtilis*	UTBSP1	85.66	[74]
B1, G1, M1		ANSB060	81.5, 81, 60	[132]
B1	*Brachybacterium* spp.	Rabbit feces	74.83	[133]
B1	*Brevundimonas* spp.	Yellow cheek feces	76.86	[133]
B1	*Cellulosimicrobium funkei*	Soil around coal factories	97	[78]
B1	*Enterobacter* spp.	Hog deer feces	76	[133]
B1	*Escherichia coli*	Strain CG1061	18–62	[80]
B1	*Klebsiella* spp.	Rabbit feces	78	[133]
B1	*Mycolicibacterium fluoranthenivorans* (formerly *Mycobacterium fluoranthenivorans*)	DSM 44556	>90	[134]
B1	*Mycolicibacterium smegmatis* (formerly *Mycobacterium smegmatis*)	N.A.	>90	[82]
B1, G1, M1	*Myxococcus fulvus*	ANSM068, Deer feces	72, 68, 64	[83]
B1	*Nocardia corynebacterioides* (formerly *Flavobacterium aurantiacum*)	DSM 12,676	74.5	[135]
		NRRL B-184	85	[136]
B1, B2M1	*Pseudomonas aeruginosa*	Grain kernels and soils	83, 47, 32	[137]
B1	*Pseudomonas stutzeri*	F4 strain, *Budorcas taxicolor* feces	90	[138]
B1	*Rhodococcus erythropolis*	Strain 4.1491	96	[139]
		ATCC 4277		[140]
		18 strains	70–100	[141]
		DSM 14303	83	[134]
B1	*Streptomyces aureofaciens*	ATCC 10762	88	[140]
B1	*Streptomyces lividans*	TK 24	86	[140]
B1	*Stenotrophomonas maltophilia*	South American tapir feces	85	[133]

**Table A3:** Aflatoxin elimination by some yeast cultures.

Toxin	Fungi	Strain	Toxin Elimination (%)	References
B1	*Aureobasidium pullulans*	H1	68.61	[142]
B1	*Candida albicans*	AA17	50.34	[142]
		AA19	64.61	
B1	*Diutina catenulata* (formerly *Candida catenulata*)	N.A.	<15	[93]
B1	*Candida krusei*	N.A.	15–60	[93]
		AUMC 8161	100	[143]
B1, B2, G1, G2	*Candida parapsilosis*	N.A.	15–72	[93]
		IP1698	99.94	[144]
B1	*Wickerhamiella sorbophila* (formerly *Candida sorbophila*)	ECF16	52.59	[142]
		ECF85	51.49	
B1	*Debaryomyces hansenii*	N.A.	15–39	[93]
M1	*Kluyveromyces lactis*	N.A.	60–69	[145]
B1	*Komagataella pastoris*	EW1	71.5	[142]
		EW3	51.5	
		EW6	50.5	
B1	*Wickerhamomyces anomalus* (formerly *Pichia anomala*)	N.A.	15	[93]
		AUMC 2674	100	[143]
AFs	*Meyerozyma guilliermondii* (formerly *Pichia guilliermondii*)	AUMC 2663	80	[143]
B1	*Pichia membranifaciens*	N.A.	40–59	[93]
B1	*Rhodotorula mucilaginosa*	various strains	52.77–70.2	[142]
B1	*Saccharomyces cerevisiae*	N.A.	10–60	[93]
		CECT 1891	- *	[146]
		A18	53	[105]
		26.1.11	48.8	[105]
		RC 016	-	[101]
		EB34	52.25	[142]
		EB57	51.12	
M1	*Saccharomyces cerevisiae*	SAFLAGER W37/70	90.3	[147]
		N.A.	81.3	[107]
		ATCC 9763	75	
B1	*Saccharomycopsis fibuligera*	N.A.	<15	[93]
B1	*Saccharomycodes ludwigii*	N.A.	40–59	
B1	*Schizosaccharomyces pombe*	N.A.	40–59	
B1	*Cutaneotrichosporon mucoides* (formerly *Trichosporon mucoides*)	N.A.	<15	
B1	*Zygosaccharomyces bailii*	N.A.	15–39	

* no data.
